# Technical Considerations of Giant Right Coronary Artery Aneurysm Exclusion

**DOI:** 10.1155/2016/3795640

**Published:** 2016-11-27

**Authors:** James Barr, Metesh Nalin Acharya, Antonios Kourliouros, Shahzad Gull Raja

**Affiliations:** Department of Cardiothoracic Surgery, Harefield Hospital, Hill End Road, Harefield, Middlesex UB9 6JH, UK

## Abstract

Giant coronary artery aneurysms are rare clinical entities. We report the case of a 49-year-old man who presented with dyspnoea and exertional chest pain. Investigations confirmed an aneurysmal right coronary artery measuring 4 cm with a fistulous communication to the right atrium. Following right atriotomy, the fistula was oversewn and the aneurysmal right coronary artery ligated at its origin and at several points along its course. A saphenous vein graft was anastomosed to the posterior descending artery. Persistent ventricular fibrillation occurred upon chest closure, attributed to ischaemia following ligation of the aneurysmal coronary artery. Emergent resternotomy and internal defibrillation were successfully performed. The sternum was stented open to reduce right ventricular strain and closed the following day. The patient made an unremarkable recovery. We here address the technical challenges associated with surgical repair of right coronary aneurysms and the physiology and management of potential complications.

## 1. Introduction

Coronary artery aneurysms are rare and may present with thromboembolic or ischaemic phenomena, rupture, or compressive symptoms [1, 2]. We present the case of a symptomatic 49-year-old male with a 4 cm right coronary artery (RCA) aneurysm communicating with the right atrium (RA). Technical aspects of the procedure and potential pitfalls of surgical correction are discussed in view of the limited published literature on this subject.

## 2. Case Presentation

**Figure 1:**
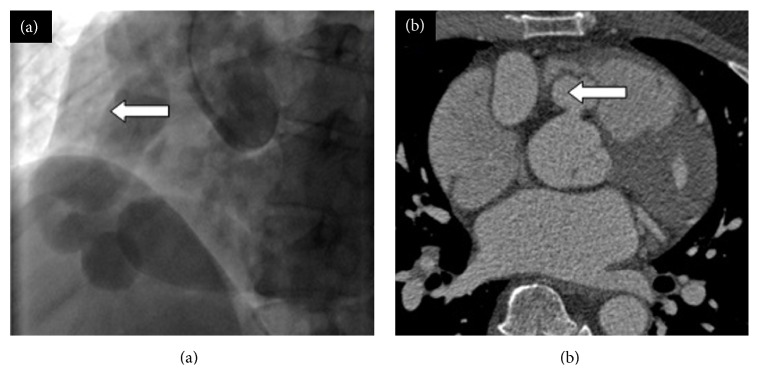
(a) Coronary angiogram demonstrating the aneurysmal right coronary artery on right coronary injection (arrow). (b) Computed tomography (CT) coronary angiogram showing the aneurysmal right coronary artery arising from the normal position on the aorta (arrow).

**Figure 2:**
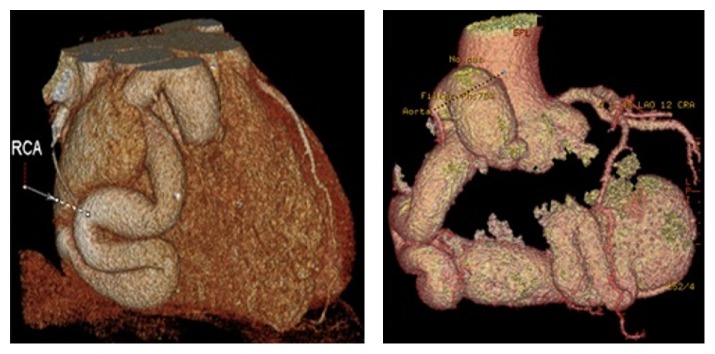
Three-dimensional reconstructions of CT images demonstrating the course of the aneurysmal right coronary artery on the surface of the heart.

A 49-year-old man with a background of hypertension and hypercholesterolaemia presented with dyspnoea and exertional chest pain. Coronary angiography (Figure 1(a)) demonstrated an RCA aneurysm in a right-dominant coronary system. Computerised tomographic (CT) coronary angiography (Figure 1(b)) and three-dimensional reconstruction of CT images (Figure 2) further characterised the aneurysmal RCA to be arising from the normal position on the aorta with fistulous connection to the RA. Transesophageal echocardiography demonstrated preserved biventricular function with a mildly dilated right ventricle (RV) and no significant valvular abnormalities. The coronary sinus was dilated to 40 mm but agitated saline (bubble) test was negative for persistent left superior vena cava. Following multidisciplinary discussion, informed consent was obtained for surgical intervention on symptomatic and prognostic grounds.

**Figure 3:**
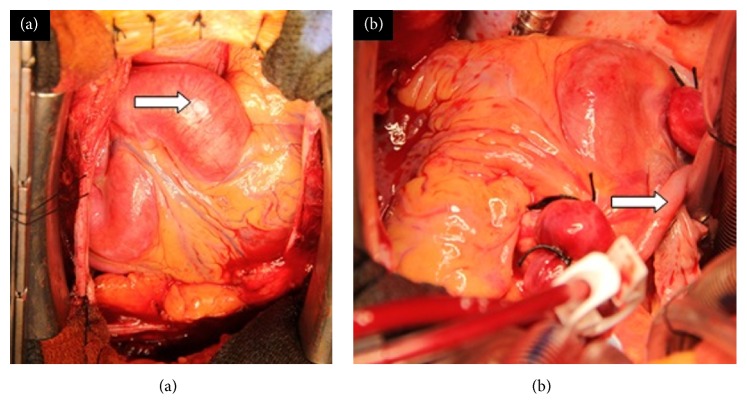
(a) Intraoperative view of right coronary artery aneurysm (arrow). (b) The right coronary artery aneurysm following ligation with saphenous vein graft to posterior descending artery in situ (arrow).

At operation, the aneurysmal RCA was exposed following median sternotomy (Figure 3(a)). It was dilated at 4 cm and tortuous with a normal course along the anterior atrioventricular groove descending down towards the crux but extending further and then turning back to drain into the RA just anterior to the insertion of the inferior vena cava. The posterior descending artery (PDA) arose from the distal aspect of the artery. Cardiopulmonary bypass was established with standard ascending aortic and bicaval cannulation, with snares around the venous cannulae. Initially, 1.5 litres of antegrade cold-blood cardioplegia was delivered into the aortic root, followed by intermittent infusion at 20-minute intervals. Following right atriotomy, two fistulous connections identified between the aneurysmal RCA and the RA were oversewn with a 4-0 polypropylene suture followed by closure of the right atriotomy. A bypass graft from the ascending aorta to the PDA was performed with a segment of long saphenous vein. The RCA was flush ligated at its ostium and multiple ligations were performed downstream with heavy silk ties (Figure 3(b)). The patient was weaned off cardiopulmonary bypass with stable haemodynamics without any inotropic support.

On skin closure, the patient developed unexpected ventricular fibrillation (VF). External direct-current cardioversion (DCCV) was successfully performed, although the patient then developed recurrent episodes of VF. A lignocaine infusion was commenced alongside emergent resternotomy and internal DCCV with eventual restoration of sinus rhythm. Satisfactory blood flow was observed in the saphenous vein graft, although right ventricular and septal hypokinesia were demonstrated on transesophageal echocardiography, consistent with right ventricular stunning. The right pleura was opened and the sternum stented open to relieve any potential cardiac compression, and milrinone and noradrenaline infusions were commenced. The patient was transferred to intensive care with stable haemodynamics. Following uncomplicated sternal closure, the patient was extubated on the following day. He made an uneventful recovery and was discharged home seven days after surgery.

## 3. Discussion

A coronary artery is defined as aneurysmal when the diameter of the vessel is 1.5 times that of the patient's largest coronary artery. It is reported in 1.5–4.9% of angiographic studies and 1.5% of pathological studies [1, 2]. Giant coronary artery aneurysms are those exceeding 2–5 cm in size and are even rarer with an incidence of 0.02% [2]. They are most commonly found in the right coronary artery, followed in incidence by the left anterior descending, left mainstem, and left circumflex arteries [3]. Complications include rupture, thromboembolism, and cardiac compression. More infrequently, fistulation into a cardiac chamber can occur [4].

Coronary artery aneurysms are classified as acquired or congenital. Approximately 50% of acquired coronary artery aneurysms are associated with atherosclerosis [5]. Nonatherosclerotic aetiologies of coronary artery aneurysm include Kawasaki disease, connective tissue diseases such as Marfan's syndrome, autoimmune diseases such as Takayasu's disease, polyarteritis nodosa and scleroderma, infections, recreational drug abuse, blunt chest trauma, and iatrogenic injury during percutaneous coronary interventions [1, 5]. In the present case, it is unclear whether pathology represented a coronary artery aneurysm that is fistulated into the RA or a congenital fistula with subsequent coronary dilatation.

Our patient had right-dominant circulation and a PDA measuring 1.25 mm, necessitating bypass grafting on exclusion of the aneurysmal RCA. However, other smaller vessels commonly supplied by the RCA, such as branches to the sinoatrial and atrioventricular nodes and marginal branches, lost supply from the aneurysmal RCA following its ligation. We postulate that acute ischaemia following aneurysm ligation, in the territories supplied by these important branches, may have precipitated VF in our patient. As VF occurred during chest closure, we employed stenting of the chest to minimise the right ventricular strain and oedema that had already occurred. We speculate that early chemical suppression of the arrhythmia, correction of electrolyte and acid-base abnormalities arising as the result of oxidative stress and, eventually, collateralisation contribute to the restoration of right heart electromechanical function.

There is no evidence-based consensus on the optimal management strategy of giant coronary artery aneurysms, and management varies according to the underlying aetiology, size and location of the aneurysm, patient symptoms, and associated comorbidities. For small, asymptomatic coronary artery aneurysms, conservative medical management with aggressive modification of cardiovascular risk factors, antiplatelet therapy, and addition of anticoagulant drugs to reduce thromboembolic complications may suffice. Surgical intervention consisting of aneurysm resection or ligation, with or without concomitant coronary artery bypass, is usually performed for symptomatic patients, those with larger coronary artery aneurysms producing mass effect, or high-volume shunts from fistulas. Thrombosis of the aneurysm leading to myocardial infarction or embolisation is additional indication for intervention. Rupture of coronary artery aneurysms, whilst rare, is more common with congenital aneurysms than those secondary to atherosclerosis [4]. Although some authors advocate surveillance of giant coronary aneurysms and associated fistulae [6], it is difficult to calculate the risk of complications against those of surgery. Patients with prohibitive surgical risk may alternatively be offered percutaneous treatment with covered stent placement for aneurysm exclusion. Coil embolisation of coronary artery aneurysm has also been described [7]. The overall 5-year prognosis is reported at 71% [1].

In the present case, we were concerned that the high-volume shunt could lead to RV failure as RV dilatation was already evident on preoperative echocardiography. Limited information exists about the timing of surgical intervention in coronary artery aneurysms. We therefore recommend surgery with a combined aneurysm exclusion/revascularisation strategy, if the risk is acceptable, and with anticipation of potential complications and their management.
